# Tunable X-ray dark-field imaging for sub-resolution feature size quantification in porous media

**DOI:** 10.1038/s41598-021-97915-y

**Published:** 2021-09-16

**Authors:** Benjamin K. Blykers, Caori Organista, Matthieu N. Boone, Matias Kagias, Federica Marone, Marco Stampanoni, Tom Bultreys, Veerle Cnudde, Jan Aelterman

**Affiliations:** 1 Pore-Scale Processes in Geomaterials Research Group (PProGRess), Department of Geology, Ghent University, Krijgslaan 281/S8, 9000 Ghent, Belgium; 2 Ghent University Centre for X-Ray Tomography (UGCT), Proeftuinstraat 86/N12, 9000 Ghent, Belgium; 3 Swiss Light Source, Paul Scherrer Institute, 5232 Villigen, Switzerland; 4 Institute for Biomedical Engineering, University and ETH Zurich, 8092 Zurich, Switzerland; 5 Department of Physics and Astronomy-UGCT, Ghent University, Proeftuinstraat 86, 9000 Ghent, Belgium; 6 Environmental Hydrogeology, Department of Earth Sciences, Utrecht University, Princetonlaan 8a, 3584 CB Utrecht, The Netherlands; 7 IPI-TELIN-IMEC, Ghent University, Ghent, Belgium

**Keywords:** Imaging techniques, Structural properties, Applied physics

## Abstract

X-ray computed micro-tomography typically involves a trade-off between sample size and resolution, complicating the study at a micrometer scale of representative volumes of materials with broad feature size distributions (e.g. natural stones). X-ray dark-field tomography exploits scattering to probe sub-resolution features, promising to overcome this trade-off. In this work, we present a quantification method for sub-resolution feature sizes using dark-field tomograms obtained by tuning the autocorrelation length of a Talbot grating interferometer. Alumina particles with different nominal pore sizes (50 nm and 150 nm) were mixed and imaged at the TOMCAT beamline of the SLS synchrotron (PSI) at eighteen correlation lengths, covering the pore size range. The different particles cannot be distinguished by traditional absorption µCT due to their very similar density and the pores being unresolved at typical image resolutions. Nevertheless, by exploiting the scattering behavior of the samples, the proposed analysis method allowed to quantify the nominal pore sizes of individual particles. The robustness of this quantification was proven by reproducing the experiment with solid samples of alumina, and alumina particles that were kept separated. Our findings demonstrate the possibility to calibrate dark-field image analysis to quantify sub-resolution feature sizes, allowing multi-scale analyses of heterogeneous materials without subsampling.

## Introduction

Porous media are omnipresent in our lives, ranging from batteries^1^ and bones^2^ to building materials^3^. The microstructural properties of these materials have a large impact on the objects’ performance, mechanical strength and durability. Understanding the features of the pore space therefore drives the development of better materials. The bulk properties of porous materials can be characterized using physical laboratory-based methods, including the capillary uptake of water, mercury intrusion porosimetry (MIP), or gas expansion pycnometry. A complementary approach is to visualize the pore space with imaging techniques such as optical and electron microscopy, X-ray and neutron imaging, and ultrasound imaging^4^. Each of these techniques has its respective advantages and disadvantages with respect to resolution, sample preparation, destructiveness, output format, etc. Whilst physical laboratory measurements primarily give information averaged over the entire sample, visualizing the pore space has the added benefit of showing individual pores, displaying their size, shape, and connections to other pores, yet at the cost of additional computational complexity during the analysis.

In the last 20 years, high-resolution computed X-ray tomography (µCT) has become an important method to non-destructively investigate the internal structure of materials in three (and even four) dimensions^5–13^. µCT is based on the X-ray attenuation properties of the materials, which depend on both their chemical composition and local density^9,14^, and can be performed with both synchrotron- and laboratory-based sources. The X-ray attenuation is described by the Lambert–Beer law^14^ that states that X-rays are attenuated as a function of the material they are propagating through.

In the reconstructed tomograms materials with sufficiently distinct compositions or densities can be differentiated, with a spatial resolution determined by the geometry of the setup and instrumentation^15^. A rule of thumb is that the best achievable resolution is about three orders of magnitude times smaller than the field-of-view (FOV)^14^. This results in a trade-off between the FOV and spatial resolution for both synchrotron and lab-based setups, implying that at the highest resolution, the imaged volume is not representative for materials that exhibit multi-scale structural heterogeneity (e.g. natural stones).

Ideally, for a pixel-limited imaging system, every voxel in a µCT volume contains the local linear attenuation coefficient, resulting from an average of all structures located within that voxel. An air-filled pore smaller than a voxel will cause the reconstructed attenuation coefficient to shift to lower values, while the reverse will happen for dense micro-inclusions^16^. These so-called partial volume effects can indicate the presence of sub-resolution features (e.g. micro-pores) in micro-CT images. Sub-resolution pores can also be detected by subtracting images before and after contrasting agents such as cesium chloride (CsCl) or potassium iodide (KI) have been added^17,18^. However, disadvantages of this method include (1) possible alteration of the material by the agent, (2) false conclusions as agents do not always penetrate the entire pore space, and (3) the technique no longer being non-destructive due to the introduction of agents, and (4) the need for a more complicated set-up^19^.

Characterizing samples with sub-resolution pores is challenging: ignoring these pores results in an underestimation of the porosity and pore connectivity, but including them requires either assumptions and generalizations, and/or data from other methods such as SEM or MIP^20^. A reliable correlation of these different datasets is also far from straight-forward. Sub-resolution pores play an essential role in various pore-scale processes such as imbibition and freeze–thaw weathering of building stones^21,22^.

In the last 15 years, X-ray and neutron dark-field imaging (DFI) have been explored as techniques to overcome this trade-off between spatial resolution and FOV. These measurements can be performed using grating interferometry (GI)^23^, speckle-based imaging^24^ or edge-illumination^25^. Building on the recent advances of Kagias et al.^26^, we explore X-ray DFI using GI for feature size quantification. GI was originally developed to obtain phase contrast images, but soon after its implementation the dark-field imaging mode was discovered, together with its promising abilities in a wide range of applications^27–29^: detecting micro-calcifications in breast tissue^30^, identifying explosives^28^, detecting micro-cracks in carbon fiber reinforced polymers^31,32^ or defects in welded aluminum^33^. DFI has also been applied to image water transport in hardening cements^19^.

DFI is based on the X-ray scattering behavior of a material, and the obtained contrast depends on the material’s unresolved microstructure. It is sensitive to variations in the electron density on a length scale from the µm-scale down to the nm-scale^28^, thereby giving complementary structural information on features with sizes down to two orders of magnitude lower than the image resolution achieved using (conventional) µCT^27^.

### Experimental setup

**Figure 1:**
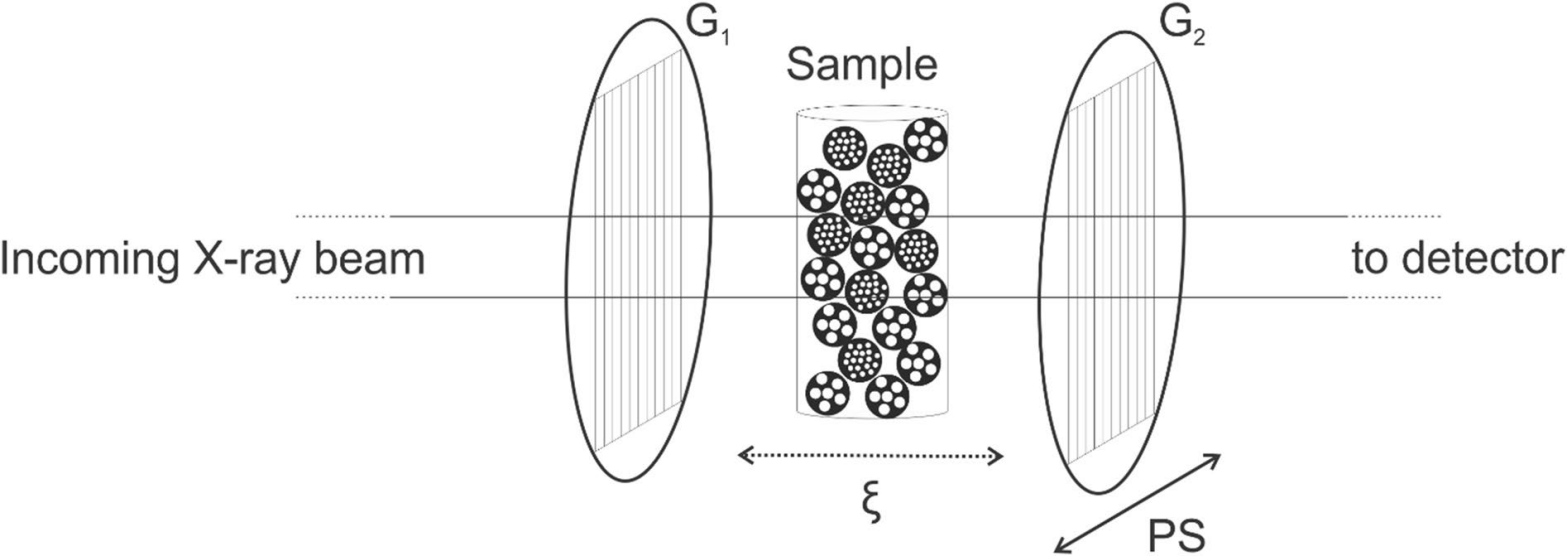
Schematic X-ray grating interferometry setup. Compared to conventional µCT, two gratings (G_1_ and G_2_) are added. To change the correlation length ($\upxi$) the sample can be moved back and forth in the direction of the beam. Phase stepping (PS) involves moving G_2_ perpendicular to the beam, resulting in an intensity oscillation at each detector pixel. Dimensions not to scale.

Relative to conventional absorption µCT, DFI acquisition in the GI approach is performed by adding two gratings to the setup, usually one phase grating and one absorption grating (Fig. 1).

**Figure 2:**
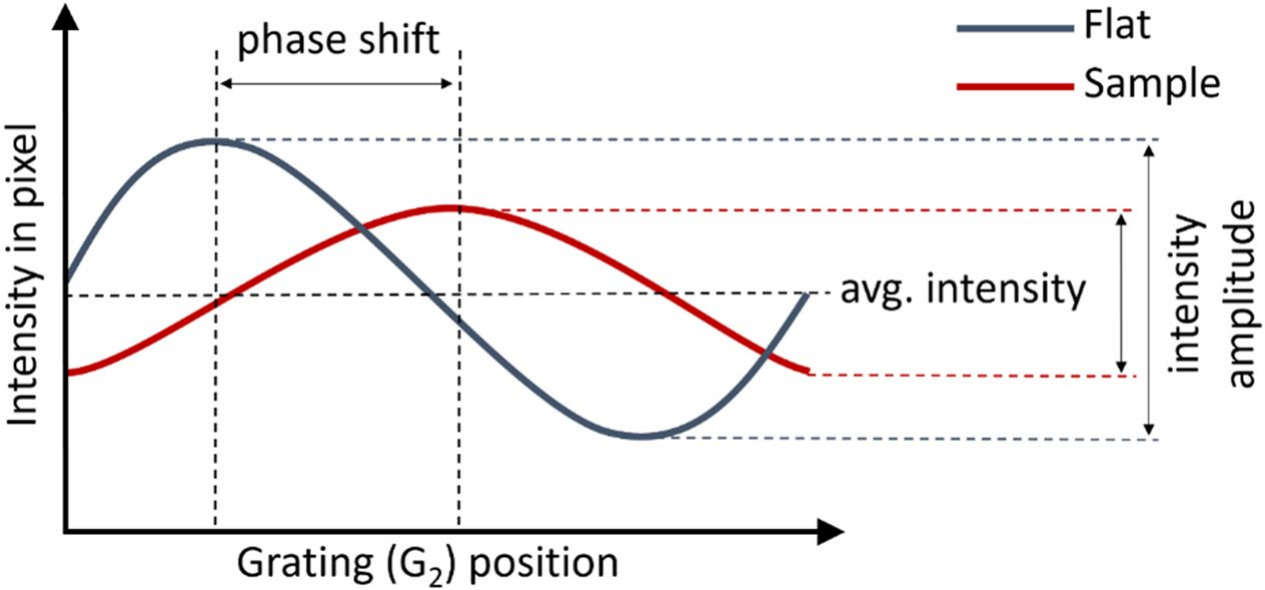
The phase stepping curve for one pixel plots the intensity oscillation measured in that pixel as a function of the distance moved by G_2_ parallel to G_1_, perpendicular to the X-ray beam. ‘Flat’: no sample is placed in the grating interferometry setup, ‘Sample’: sample is placed in the setup.

The first grating (G_1_) generates an interference pattern with a period in the micrometer range, which can usually not be resolved directly by the detector. The second grating (G_2_) has a period equal to the period of the interference pattern. To sense this pattern, G_2_ is moved perpendicular to the beam over a distance equal to the period of G1—so-called phase stepping (PS), leading to an intensity oscillation at each detector pixel^26^. By analyzing this intensity as a function of the position of G_2_, a phase stepping curve can be constructed for each pixel (Fig. 2). Based on this curve, three different types of images can be retrieved: absorption images (based on the average intensity), differential phase contrast images (based on the phase shift), and dark-field images (based on the intensity amplitude). These images are the result of X-rays being absorbed, refracted and scattered, respectively, by the sample.

### Dark-field analysis

To extract the dark-field signal or the dark-field extinction coefficient (DFEC), the so-called visibility $v$ (i.e. intensity amplitude, Fig. 2) without the sample present, is compared to the visibility ${v}^{^{\prime}}$ with the sample mounted. These visibility values are directly linked to the autocorrelation function $R\left(\upxi \right)$ of the sub-resolution structure through the following equation:

1 $${\text{DFEC}}=-\frac{1}{T}\mathit{ln}\frac{{v}^{^{\prime}}}{v}=\frac{4{\uppi }^{2}}{{\uplambda }^{2}}\left(R\left(0\right)-R\left(\upxi \right)\right)$$

with $T$ the sample thickness, $\xi$ the correlation length of the measurement and $\uplambda$ the constant X-ray wavelength^34^. Kagias et al.^26^ demonstrated the tunability of X-ray grating interferometry, meaning that the image contrast can be tuned to a particular correlation length ($\xi$) (this provides length scale sensitivity) by changing the positions of the gratings or the sample along the X-ray beam axis. When the positions of the gratings are fixed (as was the case in this research), the correlation length ($\xi$) only depends on the position of the sample, according to the following formula:

$$\xi = \frac{\lambda \cdot {L}_{s}}{p}$$

With $\lambda$ the energy of the source, ${L}_{s}$ the distance between the sample and the second grating, and $p$ the period of the interference fringe at the detector plane^26^.

### Proposed method

It has already been shown that DFI can detect sub-resolution features qualitatively^19,26,28,33–40^, but until now, methods demonstrating quantification of the size of the unresolved porosity have not been presented in published research. In this research, we present a method to estimate the size of the unresolved pores in a material, based tunable grating interferometry performed at the TOMCAT beamline of the Swiss Light Source (Paul Scherrer Institut). We validate this quantification by showing that the method can identify mixed grains of two porous materials with an identical composition (alumina, Al_2_O_3_) and very similar density but a different microstructure. The two types of grains had nominal pore sizes of 50 nm and 150 nm, respectively. Pores of this size are well below the resolution (isotropic voxel size of 1.62 µm). The sample was scanned at 18 correlation lengths ranging from 24.83 to 434.60 nm, covering the pore size range in the sample.

To establish the robustness and repeatability of the proposed quantification, we have applied the same workflow on two additional samples: grains of the alumina that were not mixed, and larger single subsamples of the alumina were imaged at the same correlation lengths (details on the sample preparation can be found in the materials section, Fig. 13). One sample was also imaged with µCT at higher resolution for comparison (isotropic voxel size of 0.65 µm). Establishing the robustness of DFI-based pore size quantification is an important step towards the accurate quantification of sub-resolution features and overcoming the field-of-view—resolution trade-off, potentially enabling a multi-scale µCT analysis without requiring very high-resolution imaging typically associated with synchrotron sources.

### Pore size quantification

The autocorrelation function, as it appears in Eq. (1), is a function of the autocorrelation length $\upxi$ defined in Eq. (2) (cfr. “Methods” section). It expresses the correlation between a signal and the shifted version of the same signal, shifted by this autocorrelation length. The signal ${\upchi }_{\mathrm{f}}\left(x,y\right)$, in this case, represents the fine part of the material’s complex refractive index^26,34^. For a non-periodic sub-resolution structure (i.e. the fine part of the material’s complex refractive index), the autocorrelation function is a monotonous negatively sloped function because the material structure will decreasingly resemble itself after larger and larger spatial shifts $\upxi$. The function slope is therefore characteristic of the material’s underlying structure—in our case unresolved porosity, with small pores resulting in a faster drop in autocorrelation as the correlation length increases. We therefore estimate the slope of $R\left(\xi \right)$, i.e. the autocorrelation function slope (AFS) to quantify the material’s pore size (see Supplementary Information S2 for a formal proposed model motivation).

**Figure 3:**
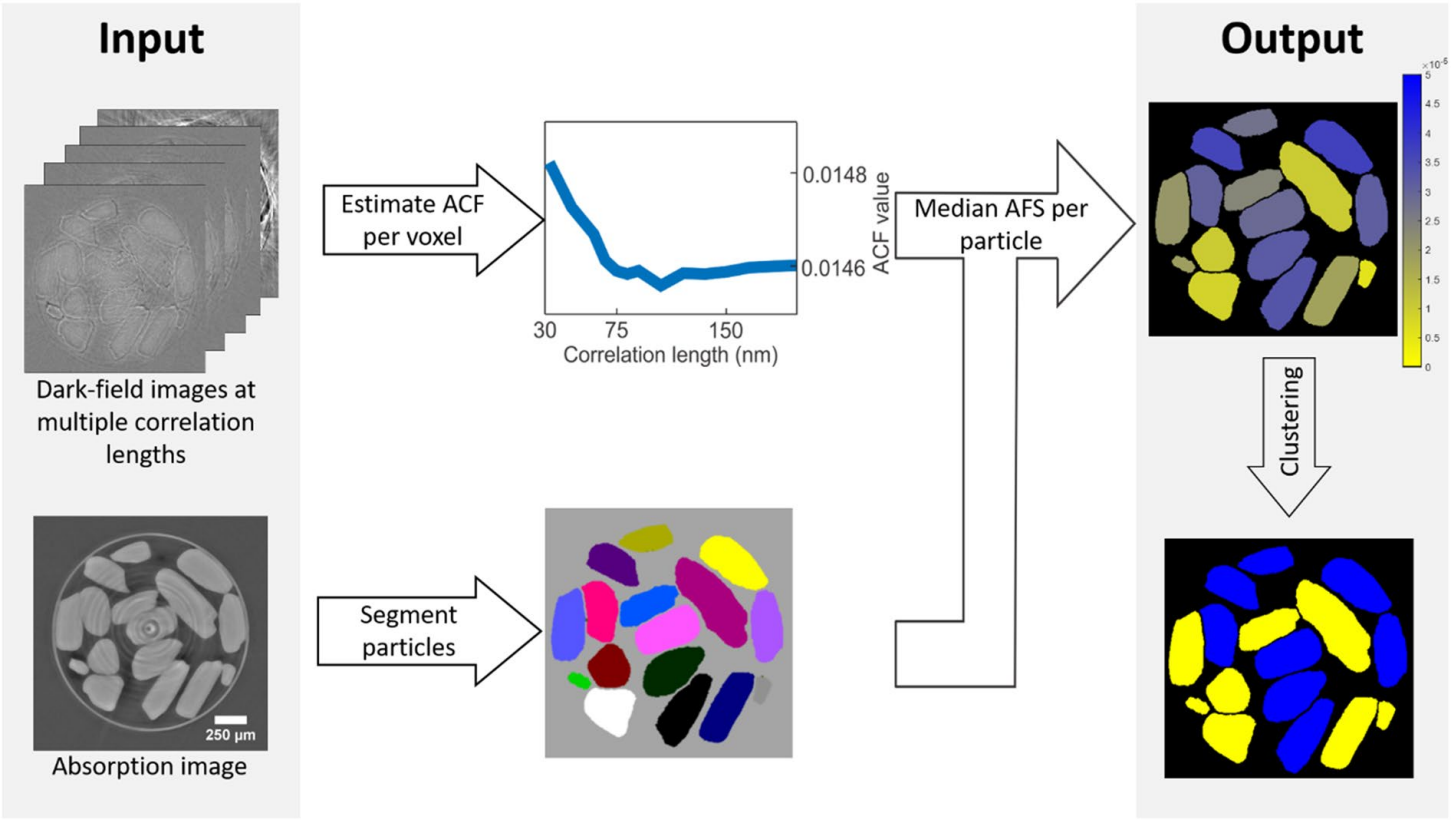
Proposed image analysis algorithm workflow. From the dark-field tomograms at multiple correlation lengths, the autocorrelation function (ACF) is estimated per voxel. Per particle, the median autocorrelation function slope (AFS) is calculated. This AFS is a proxy for the unresolved nominal pore size in the particle. Ultimately, the particles were clustered in two groups representing the particles with nominal pore sizes of 50 and 150 nm, using a k-means clustering algorithm.

**Figure 4:**
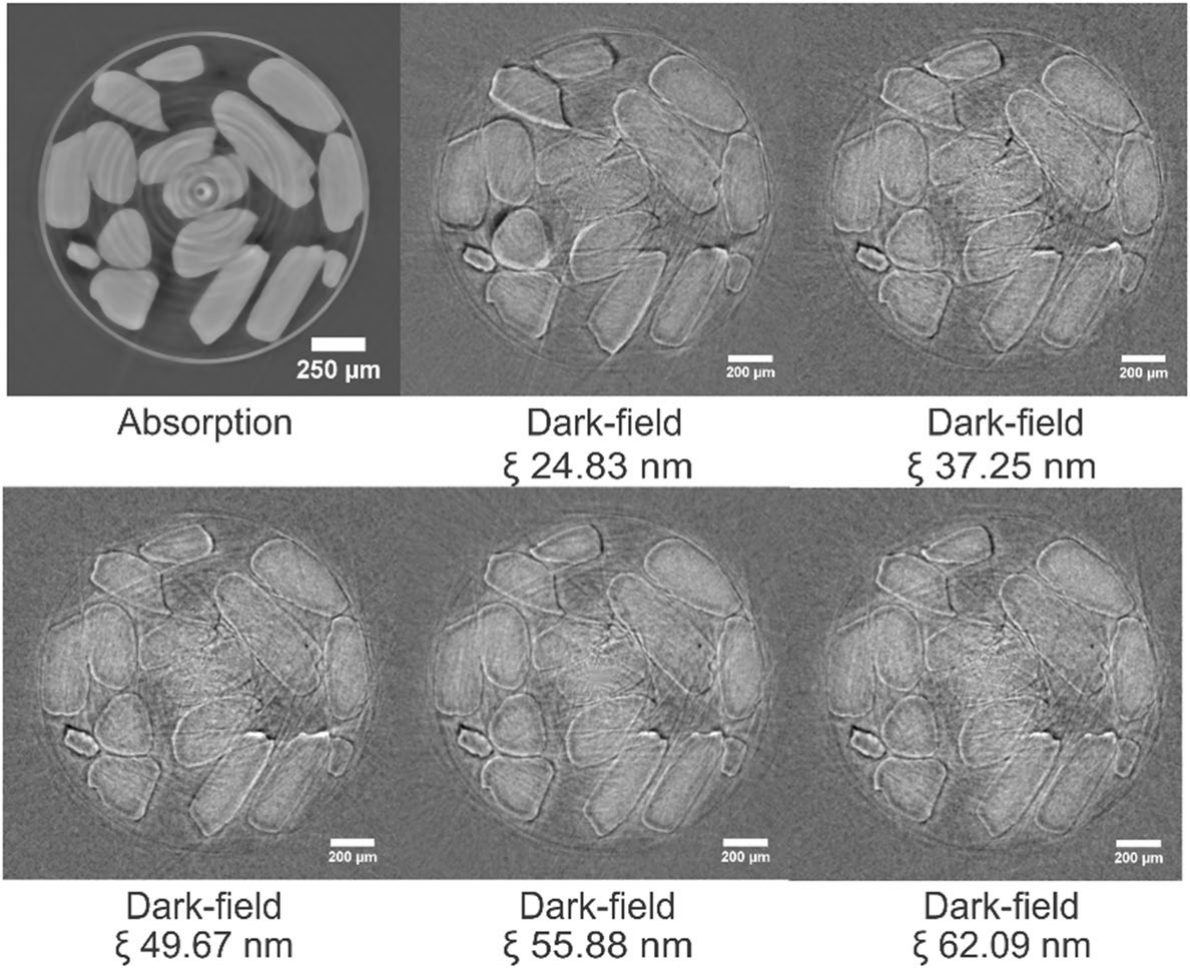
The dark-field signal changes as a function of the correlation length ($\upxi )$. The sample consists of mixed particles with either a mean nomimal pore size of 50 or 150 nm. Tomograms were obtained at eighteen correlation lengths between 24.83 and 434.60 nm. Dark-field cross-sections of the five smallest correlation lengths are show.

The first step in quantifying the AFS (Fig. 3) is to acquire dark-field images of the sample at different correlation lengths $\upxi$. This results in a series of images like the single slices shown in Fig. 4.

From the absorption images (acquired simultaneously with the dark-field images) the particles are segmented to serve as structures-of-interest. From the dark-field images, an estimate of the autocorrelation function (ACF) is extracted per voxel from the DFEC signal. Next, the median autocorrelation function slope (AFS) is calculated per segmented particle to ensure robustness against noise and artifacts. For further robustness, the edge of the segmented particle is ignored following the procedure outlined in Supplementary Information S1, intended to mitigate edge artifacts. This particle’s AFS is then a proxy for the nominal pore size in that particle.

As a last step, to validate the AFS’s ability to express pore size, the AFS values are clustered into two groups. We then associate these two groups to particles from the 50 nm disk and the 150 nm disk, respectively. More details on the applied workflow can be found in “Methods” section.

## Results

In the following sections, we will first describe the absorption and dark-field tomograms obtained using GI, which allowed us to segment the different particles, and to perform the proposed dark-field analysis for the sub-resolution pore size quantification. Subsequently, we discuss the results of the validation experiments, which are twofold: one experiment utilizes high-resolution synchrotron-based tomography of the same sample to validate the dark-field-based quantification, and the others validate the reproducibility of the dark-field signal analysis procedure by repeating it on single solid samples of the same alumina material, and on a control sample where multiple smaller alumina grains with distinct nominal pore sizes were kept separated (Fig. 13).

### Grating interferometry and image analysis

Both the absorption and dark-field dataset are acquired simultaneously, and thus inherently registered. Figure 4 shows cross-sections through the experiment dataset of the sample with mixed particles: a cross-section through the absorption dataset acquired with GI (upper left), and corresponding cross-sections through the dark-field dataset at the five smallest sampled correlation lengths.

**Figure 5:**
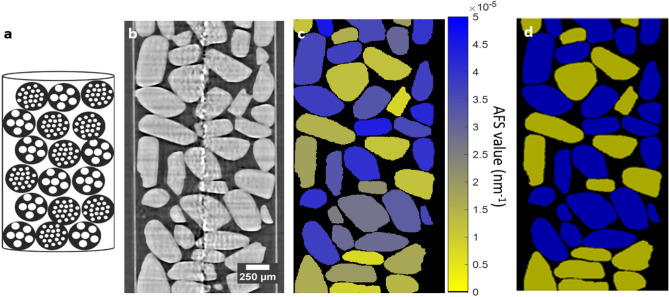
A schematic representation of the sampled with mixed particles (**a**). In (**b**) a vertical cross section through the absorption dataset is given, the vertical line in the center is due to ring artifacts. The proposed procedure derives an AFS value per segmented particle (**c**). These AFS values are then clustered to classify the 150 nm particles (yellow) from the 50 nm particles (blue) (**d**).

Figure 5b illustrates the problem under consideration: in the reconstructed absorption µCT dataset obtained with the GI (isotropic voxel size of 1.62 µm) no distinction can be made between particles with a different pore size, based on the particles’ gray value. This dataset was used to segment and label the particles. Based on the gray value segmentation performed on the absorption dataset, a region of interest (ROI) was created for every particle.

Based on analysis of the DFEC as a function of correlation length, which is equivalent with the autocorrelation function, we introduce the autocorrelation function slope (AFS). We relate this AFS value to the unresolved feature size (see Supplementary Information S2). In Fig. 5, a schematic representation of the sample is shown (a), together with a vertical cross-section through the absorption dataset (b). In Fig. 5c every particle is assigned a gray value based on its AFS. As such, the gray value is a proxy for the unresolved nominal pore size. As one of the experiments to assess its validity, we attempt to cluster the known particles based on this AFS value to assign every particle (Fig. 5d).

### Validation

In the following sections, we will validate the proposed methodology in two aspects. Firstly, we validate the resulting classification’s consistency with a classification based on a high-resolution tomogram of the same sample, in which some particles do show resolved porosity. Secondly, we validate the applied procedure robustness and reproducibility on other, similar samples. Validating this robustness is an important step towards quantifying porosity using dark-field imaging.

### High-resolution absorption tomography

**Figure 6:**
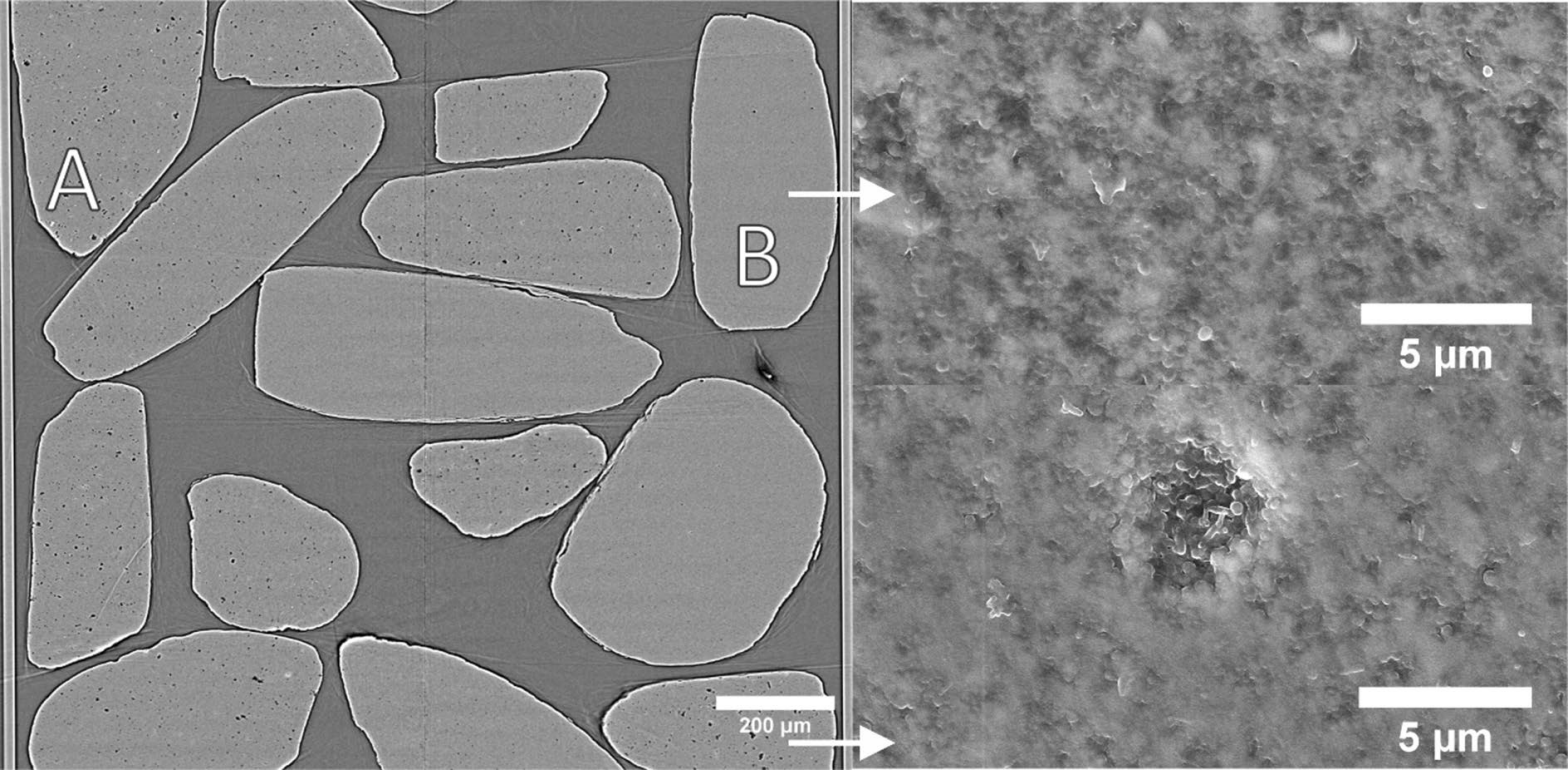
Left: cross-section through the high-resolution tomogram of the mixed particle dataset (voxel size of 0.65^3^ µm^3^). In some of the particles larger pores are visible (marked with A) whereas other particles show less or no pores at all (marked with B). Right: the white arrows point to scanning electron microscopy images of the source material with nominal pore sizes of 50 nm (top) and 150 nm (bottom). These nominal pore sizes are determined by mercury intrusion porosimetry and correspond to pore throat sizes.

**Figure 7:**
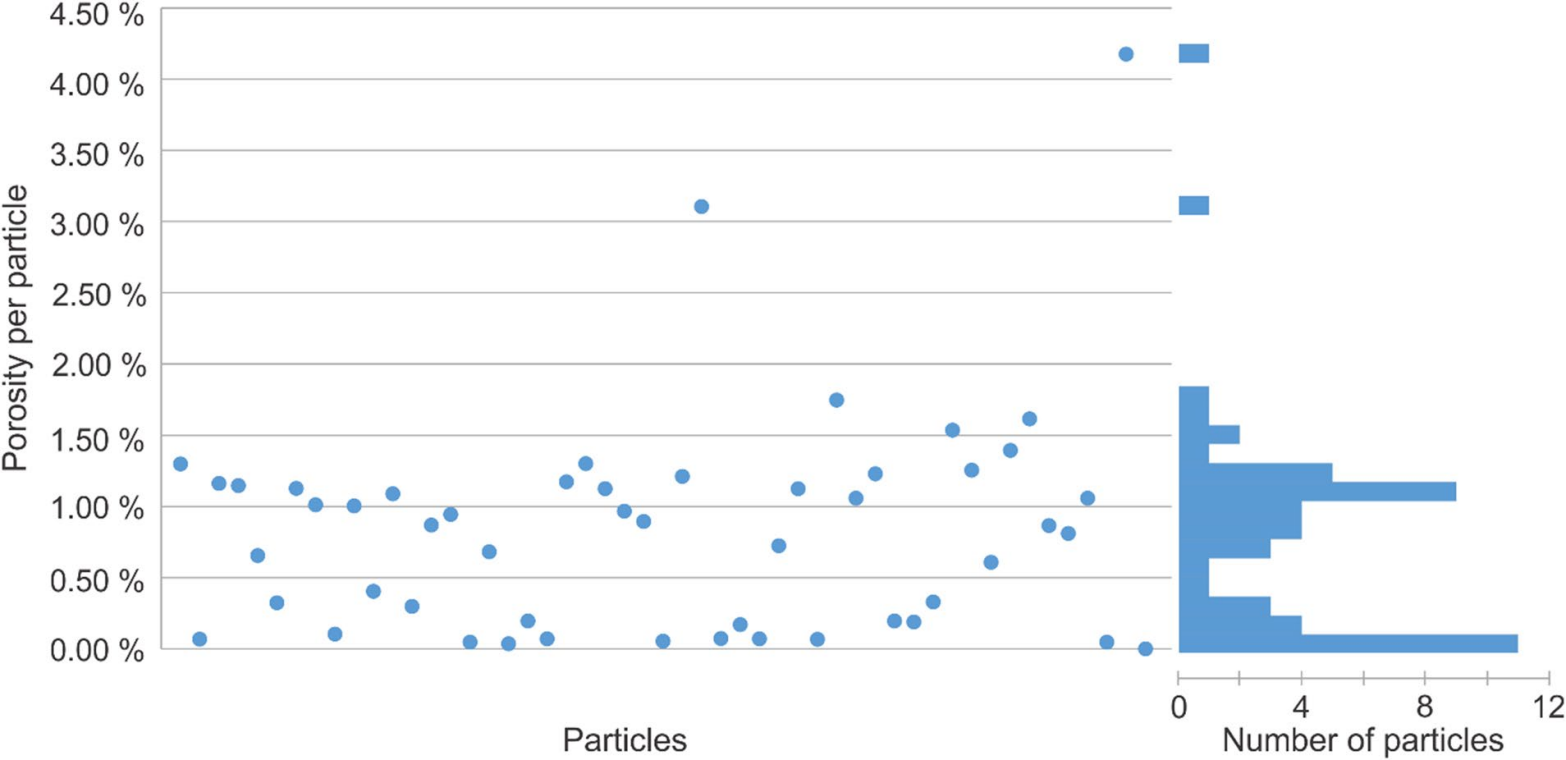
Left: scatter plot of the resolved porosity per particle. Right: histogram of particles per porosity. The bimodal distribution of porosities enabled to classify the particles in two groups.

**Figure 8:**
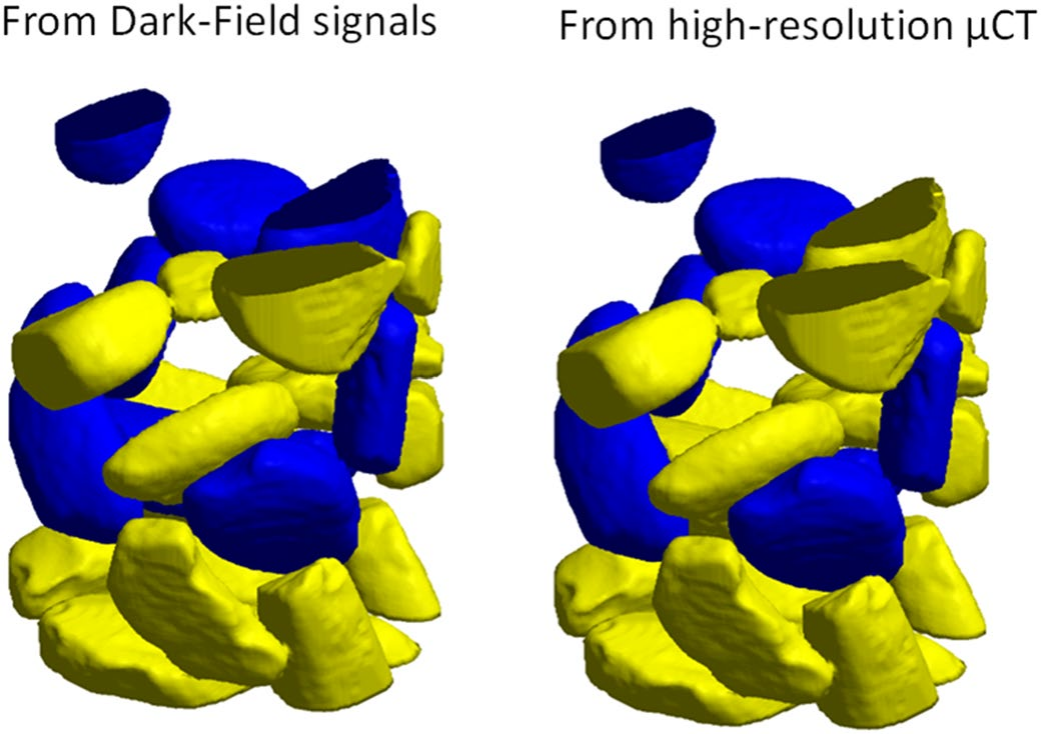
K-means classification consistency comparison. Left: particle classification based on the AFS value derived from dark-field acquisition. Right: classification based on segmentation of high-resolution μCT. Blue particles are dominated by 50 nm pores, yellow particles are dominated by 150 nm pores.

**Figure 9:**
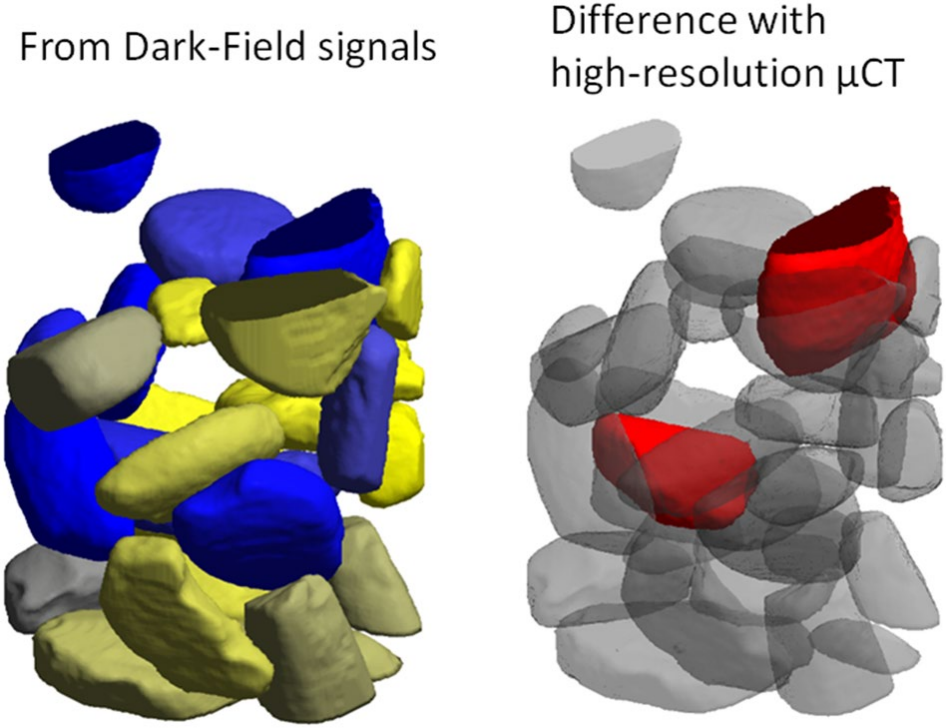
Left: classification from dark-field AFS values (analogous to Fig. 8), color saturation is now scaled by the distance from the classification decision boundary, a grayish color means “close to the decision boundary”. Right: inconsistently classified particles in red. The inconsistently classified particles seem not related to proximity to the decision boundary.

In the cross-section through the high-resolution tomogram of the mixed particle dataset (voxel size 0.65^3^ µm^3^) (Fig. 6), some particles show resolved porosity (marked with A), whilst other particles show little to no resolved porosity (marked with B). These resolved pores are much larger than the nominal pore sizes of the particles (50 and 150 nm, respectively) that are still below the resolution. The resolved porosity of every particle was determined by segmenting these pores based on their (low) gray value and dividing the pore volume by particle volume. The distribution of particles’ porosities was found to be bimodal (Fig. 7), enabling the classification of particles into two groups. Figure 8 provides two 3D renderings of these classified particles, one based on the AFS and the other on high-resolution tomography. Figure 9 shows the AFS-based classification where the color saturation relates to the distance to the classification decision bound (gray means ‘on the decision boundary’), and the two inconsistently classified particles with respect to classification based on the high-resolution tomogram. This latter classification was 94% consistent with the classification based on the AFS value, corresponding to 29 of the 31 particles that were matched between the two datasets. Notice that the inconsistently classified particles do not appear particularly close to the decision boundary.

In Figs. 8 and 9 only the particles that could be validated by high-resolution tomography, and vice versa were considered. In both the low and the high-resolution absorption dataset, mathematical morphology operations (e.g. erosion) were performed to ensure that no particles were attached to each other. This caused some particles to be ‘broken up’ into several particles in the segmentation, or even to be removed. In addition, some particles were too small to be matched to a particle of in the other dataset, based on their shape. These particles were also left out of consideration.

### Pore size quantification of solid disks and separated particles

Two other samples of the same material were considered for validation: a sample that is made up of 2 larger subsamples of the disks on top of each other, and a sample that consists of broken-down smaller particles of both disks, that were kept separated (Fig. 13). Both samples were imaged and analyzed following the same procedure as the mixed particle sample.

**Figure 10:**
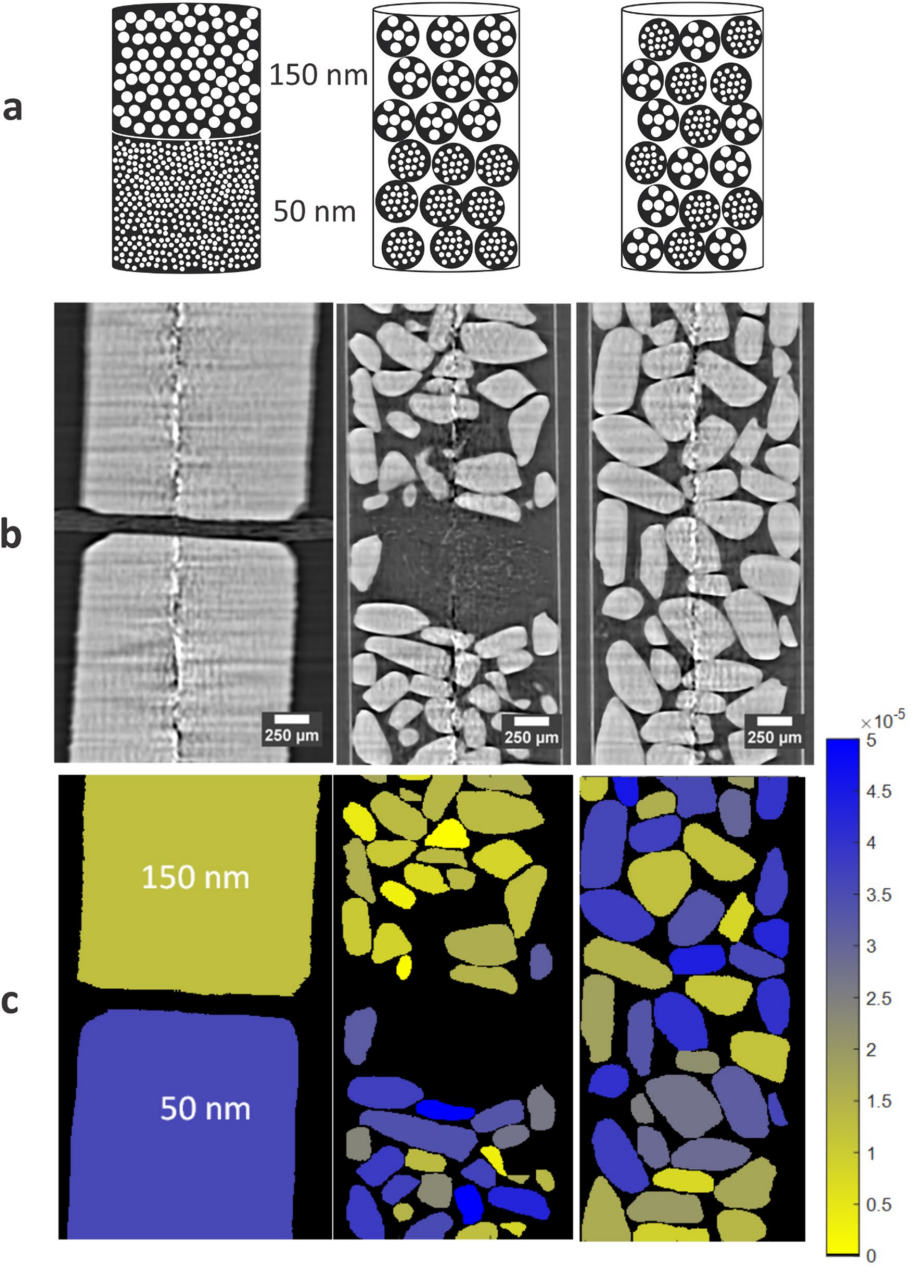
(**a**) Schematic representation of each studied sample. (**b**) Vertical cross-section through the absorption datasets, where no contrast is observed. (**c**) Segmentation where every disk or particle is assigned a color based on its AFS value.

**Figure 11:**
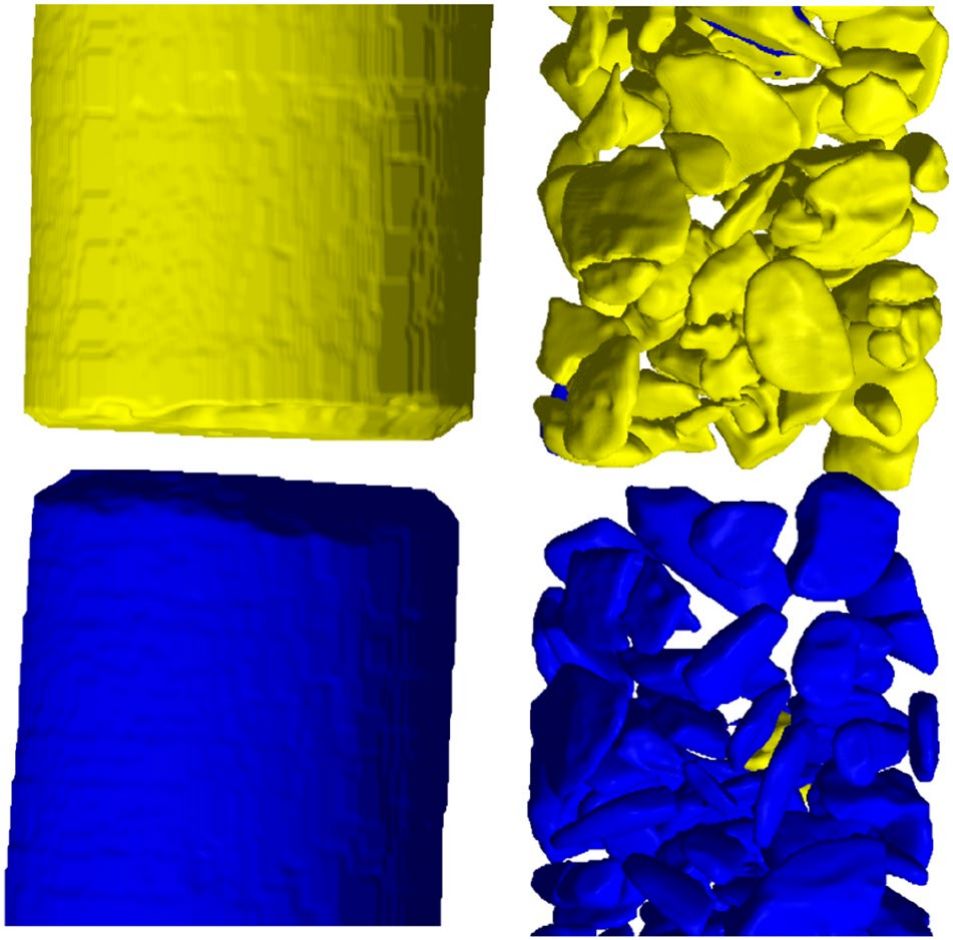
3D rendering of the AFS-based classification of the disks and separated particles. The yellow and blue colors represent 150 nm and 50 nm pores, respectively.

In Fig. 10, an overview is given of the three samples. Figure 10a shows a schematic representation of the samples consisting of disks, separated particles and mixed particles, respectively. In Fig. 10b the vertical cross-section through the absorption µCT dataset of the samples exhibit no contrast between the different material types. The vertical image artifacts are due to ring artifacts. Figure 10c illustrates the segmentation of the particles where every particle is colored according to it AFS value. The distinction between the particles with 50 nm and 150 nm pores can be seen by their difference in AFS value: higher AFS values result in blue colors, corresponding to the particles having pores of 50 nm. The yellow particles correspond to the particles with 150 nm pores. Based on these AFS values, the proposed algorithm (see “Methods” section) can classify both the disks and separated particles correctly (Fig. 11) by performing k-means clustering on the AFS values. Figure 9 (left) visualizes the certainty of classification, encoding the distance of the AFS value for a particle to the k-means decision boundary as the color saturation. In this visualization, the greyer a particle color, the less certain its classification.

**Figure 12:**
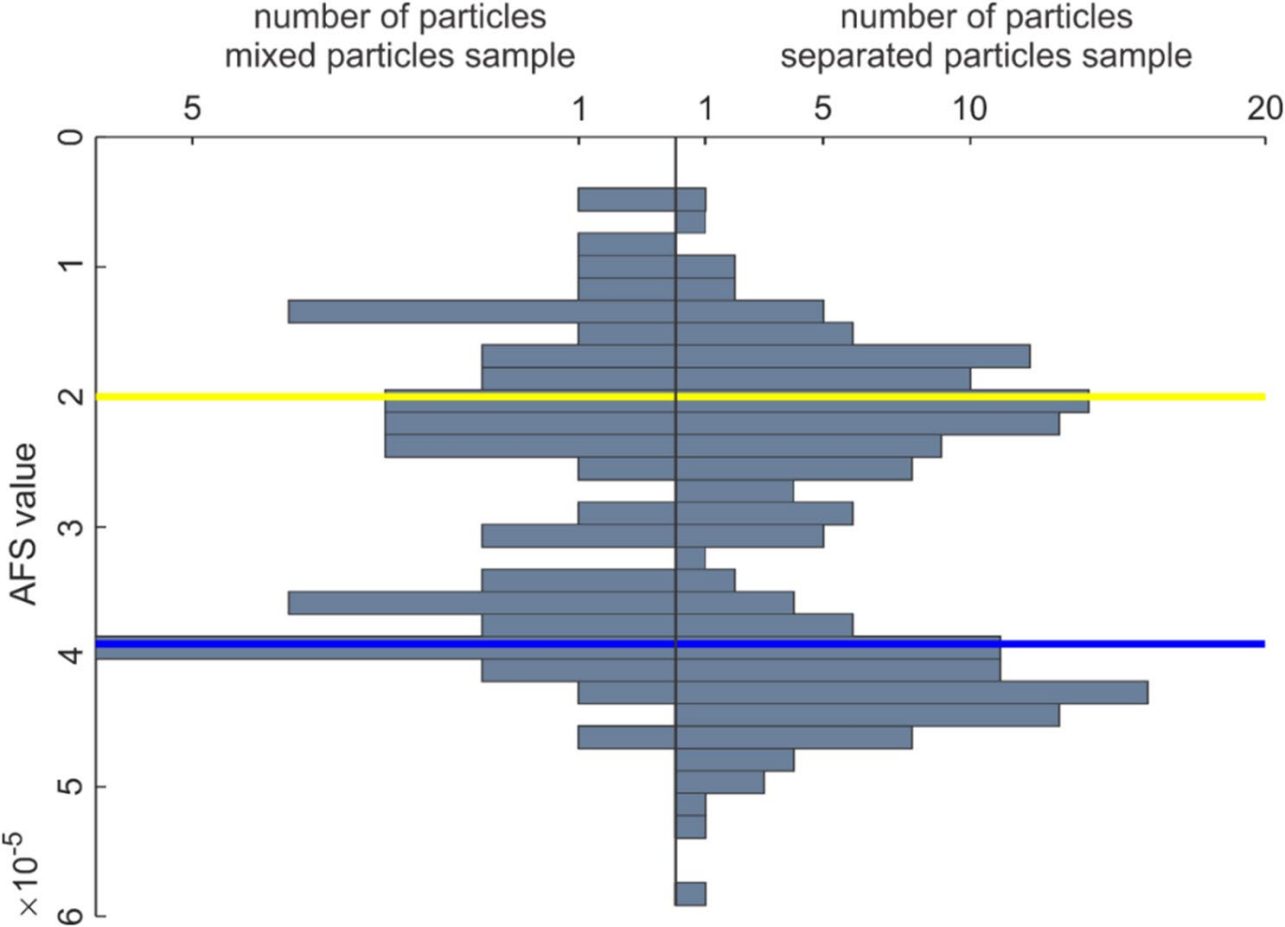
Histogram of particle AFS values for the sample with mixed particles (left) and the sample with separated particles (right). The yellow and blue lines represent the AFS value for the disk samples with 150 nm and 50 nm pores, respectively.

Figure 12 shows the AFS values for the disks (horizontal blue and yellow line), separated particles (right histogram) and mixed particles (left histogram). The AFS values were found to be consistent over the three samples, indicating the reproducibility of the applied procedure and thus the feasibility of calibrating the measurements.

## Discussion

The proposed dark-field image analysis technique computes the autocorrelation function slope (AFS) out of a per-voxel measurement of the dark-field signal at the 18 correlation lengths $(\xi )$ that were covered. We found that the five smallest correlation lengths, ranging from 24.83 to 62.09 nm, are sufficient for a reliable AFS computation and particle classification. In fact, outside of this range the dark-field signal is no longer dominated by the unresolved porosity, and, hence, no longer a reliable proxy for this unresolved pore size.

Obtaining sufficient signal-to-noise ratio for AFS estimation relies on combining $N$ voxels in a particle through the median operation. On the other hand, the dark field extinction coefficient (Eq. 1) from which the AFS is derived, lowers in signal-to-noise ratio as the sample thickness $T$ increases. Roughly approximating the DFEC noise as white Gaussian allows to formulate simple rule of thumb for the signal-to-noise ratio as function of particle size $N$ and sample thickness $T$ of:

$$SNR\sim \frac{N}{{T}^{2}}{\left(\mathit{log}\frac{{V}^{^{\prime}}}{V}\right)}^{2}.$$

In other words, there is a trade-off between the (square of the) sample size (thickness) and the size of the particles ideally used in the proposed analysis technique.

The consistency of the AFS value over the three different samples of increasing complexity (disks, separated particles, and mixed particles, Fig. 10) demonstrates both the reproducibility of the applied procedure, within the considered material class, and suggests the ability of applying DFI for quantifying sub-resolution pore sizes. One could conceive of a calibration procedure where single lumps of materials-of-interest with known pore sizes are imaged to extract their AFS value. In a second step, objects-of-interest of this material category that contain unknown porosity are then imaged and their porosity quantified by interpolating between calibrated AFS values.

Although the particles in this study were expected to have pore sizes of 50 and 150 nm with very narrow distributions, the high-resolution tomogram indicate that the actual pore sizes distribution is substantially wider, with larger pores visible in Fig. 6. A large variance in pore size may complicate linking AFS values to a single unresolved nominal pore size. Despite this added difficulty, the AFS values for both types of particles were found to be sufficiently distinct to allow a successful determination of this nominal pore size. For a material with a narrower pore size distribution, the quantification method can be expected to improve in precision.

The resolved porosity in the high-resolution tomogram enabled a secondary method to identify the different particle types. This classification was found to be 94% consistent with the AFS-based classification.

In terms of AFS precision, this study revealed an apparent standard deviation for the AFS values of 4.3e-6 nm^−1^ for the 50 nm disk sample particles and 5.5e-6 nm^−1^ for the 150 nm disk sample particles. Hypothesizing a linear relation between AFS value and pore size, this would correspond to a porosity size precision of ± 21.6 nm for the 50 nm disk particles and ± 27.7 nm for the 150 nm disk particles. For exact quantification of the unresolved pores based on the AFS values, a ground truth of the material’s pore size (distribution) is required. In future work, this ground truth can be provided by ptychographic X-ray tomography, which is able to visualize a sample’s internal structure with resolutions down to a voxel size of around 10^3^ nm^3^, or by scanning electron microscopy.

By discerning sub-resolution features with different properties (in this case different pore sizes), a significant step is made towards overcoming the trade-off between resolution and field-of view. Imaging with the dark-field modality allows porous media scientists to improve their assumptions on the sub-resolution micro-pores. It is worth mentioning that although these experiments were performed at a synchrotron facility, grating interferometry can also be performed at lab-based µCT systems. At these systems, where the achievable spatial resolution is typically lower than at a synchrotron, grating interferometry and the proposed analysis method could provide quantitative information on unresolved features, thereby overcoming the need of high-resolution imaging at a synchrotron facility, where imaging time very limited—and as such—expensive.

## Materials

**Figure 13:**
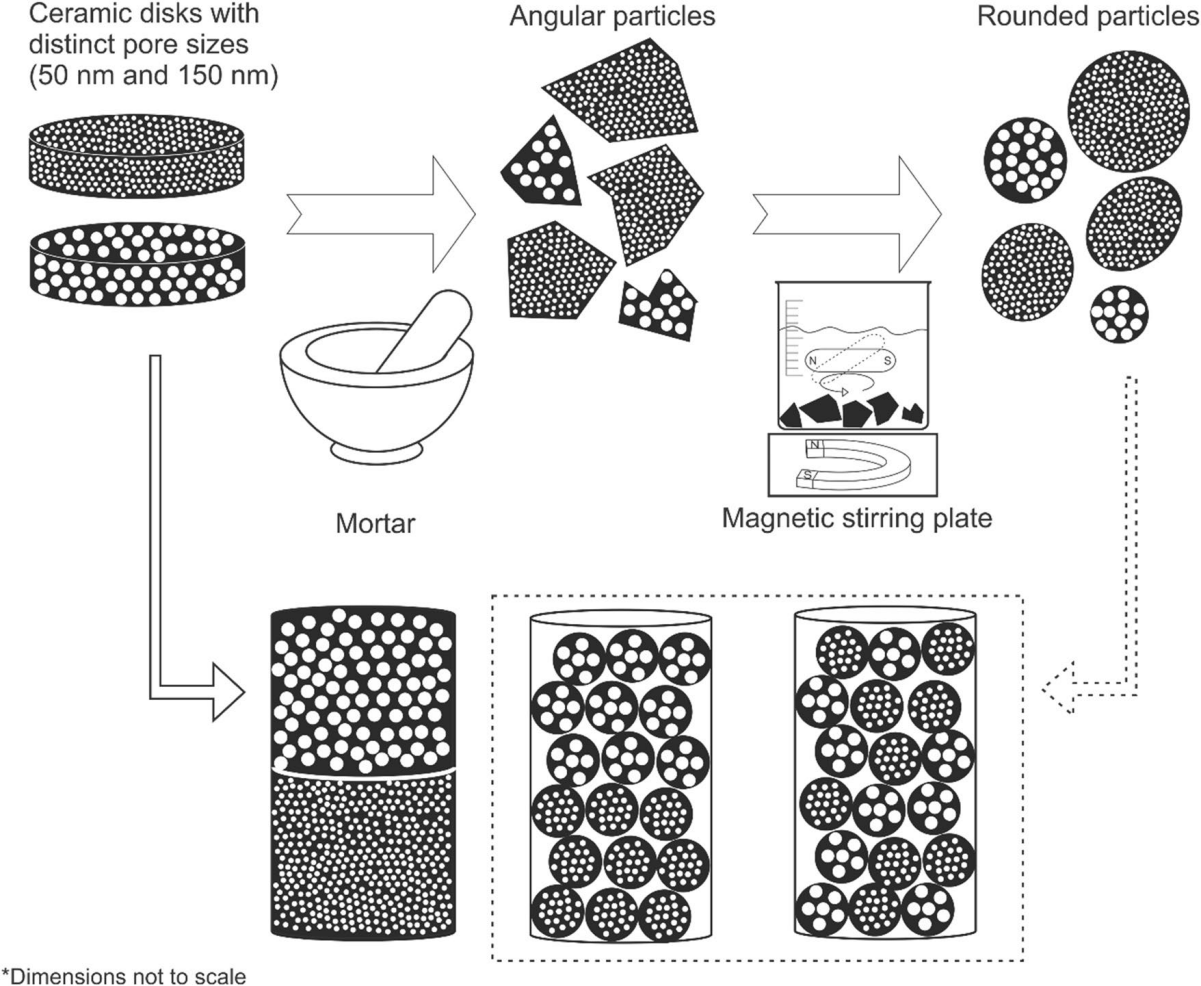
Process to create the three samples consisting of either single larger subsamples of the ceramic disks, smaller grains that were kept separated, or smaller grains that were mixed together. For the samples with the grains, ceramic disks were crushed in a mortar, and the particles were rounded and smoothed using the procedure described by Nasir et al.^41^. Black = alumina material. White = air.

Three samples were created from two ceramic disks (Cobra ceramic discs, The Netherlands), with distinct nominal pore sizes, 50 nm and 150 nm. From both these disks, a small cylinder was drilled with a diameter of 1.65 mm. A first sample consists of a stack of these two disks. A second sample is a grain pack consisting of two sets of particles, one set with pores of 150 nm on top of a set with pores of 50 nm. A third sample was made with the same type of particles, but mixed together (Fig. 13).

For the samples consisting of alumina grains, the ceramic disks were crushed to particles in a mortar. These particles were subsequently rounded by immersing them in distilled water and letting a magnetic stirrer smooth them over 24 h. The process to create the three samples, is illustrated in Fig. 13. This approach is based on the work of Nasir et al*.*^41^.

This rounding was done to have a dataset more representative for natural porous media, and to minimize edge enhancement effects: as the dark-field is sensitive to density variations, high dark-field signals occur at the edge of the sample (air-sample boundary). This signal would be amplified even more in case of a rough edge, possibly overprinting the signal arising from the particles internal structure. Lastly, the particles with a diameter between 350 and 450 µm were retained to have a grain pack with a unimodal particle size distribution.

## Methods

In the following sections, we will elaborate on how the data was acquired using grating interferometry and high-resolution tomography, and how the resulting absorption and dark-field images were processed to quantify their unresolved porosity.

### Grating interferometry

The image acquisition was performed at the TOMCAT beam line of the Swiss Light Source (Paul Scherrer Institut, Villigen, Switzerland)^42^. The interferometer was composed of one phase grating with a pitch of 4 µm and depth 21.71 µm etched in Si and an Au grating with a period of 2 µm and depth of 3.37 µm fabricated by the method described by Kagias et al.^43^ and is a $\pi$ phase shift interferometer. The grating interferometer was used in combination with a 100 µm LuAG:Ce scintillator together with pco.edge 4.2 camera and a 4 × objective. The scanning energy was 25.00 keV and the exposure time was 200 ms. The three samples were scanned at eighteen correlation lengths, ranging from 24.83 to 434.60 nm, to cover the particles’ pore sizes. Per correlation length, five phase steps were taken at equal intervals over a distance of 1.6 µm, to construct the phase stepping curve. At every phase step, 1000 projections were taken over 180°, along with 50 flat field images. The distance between the two gratings was 4.02 cm to acquire images in the first Talbot order. The isotropic voxel size in the reconstructed image, was 1.62 µm. Based on the phase stepping curve, both absorption and dark-field datasets were computed for every correlation length.

### High-resolution tomography

A high-resolution absorption tomogram of the mixed particles sample was acquired at the TOMCAT beamline (X02DA) of the Swiss Light Source, using a 100 µm LuAG:Ce scintillator together with pco.edge 5.5 camera and a 10 × objective^44^. 1501 projections were taken over 180°, as well as 50 dark images and 50 flat-field images. The scanning energy was 25.00 keV, and the exposure time 500 ms. The isotropic voxel size in the reconstructed tomograms was 0.65 µm. These high-resolution tomograms were used to help interpret the dark-field data of the mixed particle sample.

### Processing of absorption images

An absorption dataset is obtained as part of every GI acquisition. For this dataset, the disks, and particles, respectively, were segmented based on gray value. The resulting dataset was cleaned up using binary mathematical morphology operations, and the individual disks and particles were labeled. This labeled segmentation directly matches the dark-field datasets as these are both computed from the same raw data and is used to track the dark-field response of every particle over the range of correlation lengths.

The high-resolution dataset of the mixed particles was processed in the same way as the GI absorption dataset. A gray value-based segmentation followed by cleaning up the dataset with binary mathematical morphology operations, and a labeling of the particles. Every particle was manually matched to a particle in the GI dataset, based on the size, shape, and position of the particle. A manual approach was necessary as particles had moved with respect to the GI datasets.

Although the nominal pore sizes in the ceramic materials are 50 and 150 nm, respectively, some porosity is resolved at this high resolution (voxel size 0.65 µm) (Fig. 6). It is assumed that the particles with 150 nm pores will show more and larger resolved pores than the particles with 50 nm pores. The porosity per particle was determined by gray value segmentation within the particles. This porosity per particle exhibits a bimodal distribution (Fig. 7). Next, a threshold was placed between the two peaks. Particles with a lower porosity are classified as having 50 nm pores, particles with a higher porosity are classified as having 150 nm pores. This classification was used as a validation for the classification based on the AFS value.

### Processing of dark-field images

The processing aim is to quantify the relation between the inherent porosity of a sample and the dark-field extinction coefficient (DFEC)’s length scale sensitivity, i.e. the correlation length $\upxi$^26,34^. The relation is expressed through Eq. (1), which we repeat for clarity:

$${\text{DFEC}}=-\mathit{ln}\frac{{v}^{^{\prime}}}{v}=\frac{4{\uppi }^{2}}{{\uplambda }^{2}}\left(R\left(0\right)-R\left(\upxi \right)\right)$$

This autocorrelation function $R\left(\xi \right)$ is defined as the correlation between two versions of a signal that are shifted by an amount $\upxi$, expressed as a function of $\upxi$. In this case, the signal is the fine part of the material’s complex refractive index ${\upchi }_{\mathrm{f}}\left(x,y\right)$^26,34^, with x and y being perpendicular to the X-ray beam (y the phase stepping direction) and z is the direction along the X-ray beam:

2 $$R\left(\xi \right)=\frac{1}{S}{\int }_{xy}^{ }{\chi }_{f}^{ }(x,y+\frac{\xi }{2}){\chi }_{f}^{*}(x,y-\frac{\xi }{2})dxdy. $$

The higher this autocorrelation value for a particular length $\upxi$, the better the material’s refractive index resembles a version of itself that was shifted by $\upxi .$ If the structure is non-periodic, then $R\left(\xi \right)$ will therefore be a monotonous negatively sloped function of $\upxi$: the material structure will resemble less and less a version of itself after larger and larger shifts. The function slope would then be characteristic of the material’s underlying porosity structure, with small pores resulting in a faster drop in autocorrelation as $\upxi$ increases. We therefore estimate the negatively slope of $R\left(\xi \right)$, which we call the autocorrelation function slope (AFS), as a single feature-of-interest to quantify the material’s porosity scale (cfr. Supplementary Information S2 for a more rigorous motivation of this model).

The first step in quantifying the AFS is to acquire multiple dark-field images of the studied sample at different correlation lengths $\upxi$ (Fig. 4). Noise (i.e. a low signal-to-noise ratio) and edge artifacts, due to large jumps in diffraction at the edge of objects that are significantly larger than a single voxel, are apparent and these impede straightforward AFS extraction. To ensure robustness against such noise and artifacts, the proposed approach robustly extracts and aggregates a single AFS value to represent a single structure-of-interest, in this case a segmented particle. We will now explain the proposed method in detail, it follows the workflow illustrated in Fig. 3.

The “segment particles” step is explained in the previous section “processing of absorption images”, it serves to identify the voxels that correspond to the object-of-interest—the particle. Note that spatial registration is not needed due to the dark-field acquisition setup implicitly acquiring absorption images in the process. The segmentation is refined by the procedure explained in Supplementary Information S1, which is intended to exclude the edge pixel values, which are typically corrupted by edge artifacts, from consideration in the procedure.

The “estimate ACF per voxel” step extracts an estimate of the autocorrelation function (ACF) $R\left(\xi \right)$ from the DFEC signal. This step simply involves a sign inversion after ignoring the term $\frac{4{\uppi }^{2}}{{\uplambda }^{2}}R\left(0\right)$ and constant factor $\frac{4{\uppi }^{2}}{{\uplambda }^{2}}$, which are not a function of $\xi$ (but are material-dependent) and therefore would not affect relative comparison of the slope of the ACF between two samples of the same material.

**Figure 14:**
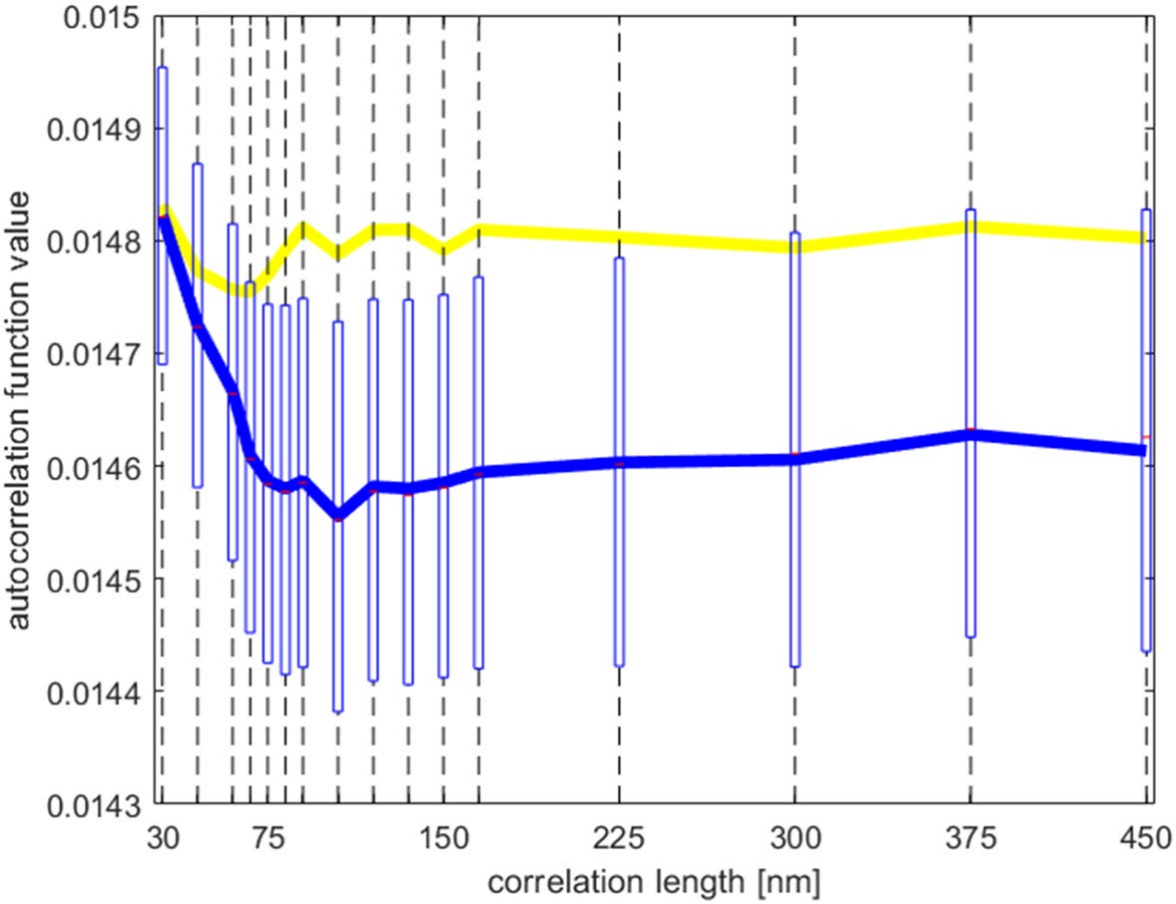
Autocorrelation function (as derived from the dark-field extinction coefficient) for the 50 nm disk sample (blue), as well first and third quartile bounds, which reveal the noisy nature of this signal. The yellow graph show the autocorrelation function for the 150 nm disk sample. Notice how the initial slope is shallower than for the 150 nm autocorrelation function.

In the “Median AFS per particle” step, the median ACF slope is calculated, considering all voxels within the particles. Figure 14 shows the median autocorrelation function for both the 50 nm and 150 nm pores disk sample in blue and yellow, respectively. The blue bars indicate the first and third quartile when considering all voxels in the disk sample. Notice how the two-part piecewise linear model explained in Supplementary Information S2 is recognizable. The procedure performs a least-squares linear fit to an increasingly large number of correlation lengths to find the steepest slope, which corresponds to the initial sloped part of the median autocorrelation function and we name the autocorrelation function slope (AFS) value. In the experiment described in the paper, the AFS values thus correspond to the least-squares fit for the range of correlation lengths between 24.83 nm and 62.09 nm (i.e. the first 5 acquired correlation lengths in the experiment).

In the “Clustering” step, the AFS values are clustered into two groups using the k-means clustering algorithm using a squared Euclidian distance optimization function. We then associate these two groups to particles from the 50 nm disk and the 150 nm disk respectively.

## Supplementary Information

Supplementary Information 1.
