# Supplementary material for: Tunable X-ray dark-field imaging for sub-resolution feature size quantification in porous media
Source: Sci Rep. 2021 Sep 16;11:18446. doi: 10.1038/s41598-021-97915-y (PMC8446041; doi:10.1038/s41598-021-97915-y)
Supplement: Supplementary file 1 — Supplementary Information 1. [file 41598_2021_97915_MOESM1_ESM.pdf]

Supplementary Information S1: Edge artifact mitigation

In dark-field imaging, the DFEC values are sensitive to scattering effects associated with high-contrast edges between structures larger than the scale of a single voxel and partial voluming. This is an undesirable effect that hinders study of the sub-resolution structure. The effect is evident from the edge contrast enhancement visible around the edge of particles in Figure 4 and Figure S1. We found the artifact is aggravated by increasing correlation length ξ. It is expected that the effect may be reduced by minimizing the difference in refractive index between the material and the medium, e.g. by using a non-gas material like in [Yang, F. et al. Advancing the visualization of pure water transport in porous materials by fast, talbot interferometry-based multi-contrast x-ray micro-tomography. Dev. X-Ray Tomogr. X 9967, 99670L (2016)]. However, it should be noted that this may limit the sample size because of increased attenuation reducing the SNR. Further research would be required to explore the practical best practices in this respect.

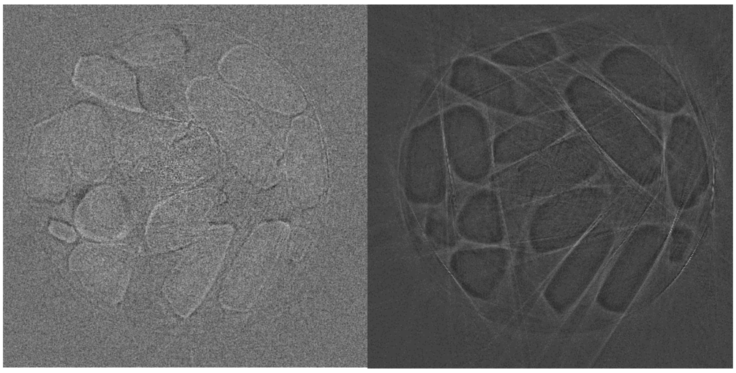

Figure S1 Illustration of the edge artifacts in DFEC signals for the mixed sample, measured for a correlation length ξ of 24.83 nm (left) and 434.60 nm (right).

The impact of this edge artifact, as well as noise in general, is visible in Figure S2: in this graph, the AFS value is extracted per voxel, rather than per object, and displayed as box plots. Per-voxel values are evidently of very low precision, making it nearly impossible of reliably distinguishing a single 50nm disk voxel from a 150nm disk voxel.

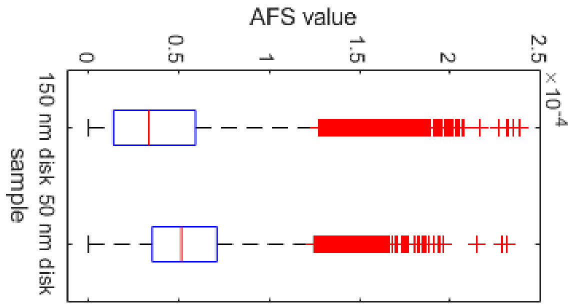

Figure S2 AFS evaluated per voxel (rather than per object) for the voxels in the disk samples and aggregated into box plots, the red crosses denote outliers.

This limited low noise robustness is the motivation for the median aggregation of segmented structure voxels in the proposed procedure. Additionally, as object edges are disproportionally affected by artifacts, our procedure includes a method of mitigating edge artifacts specifically. This is accomplished by use of mathematical morphology (erosion): specifically, the segmentation masks are iteratively eroded, so that increasingly wider layers of the edge area are ignored before the AFS value is calculated. Out of the series of AFS values thus generated, one for each erosion iteration, the lowest-variance AFS value is retained to represent the object. This is found to improve precision: Figure S3 presents boxplots of the relative increase/decrease in variance of the AFS values for the objects in the mixed sample (Fig. 13) as the amount of edge voxels that were eroded prior to the signal analysis changes, compared to the situation when no edge voxels are removed. Notice how removing 30-40% of the edge typically results in a lower-variance estimation.

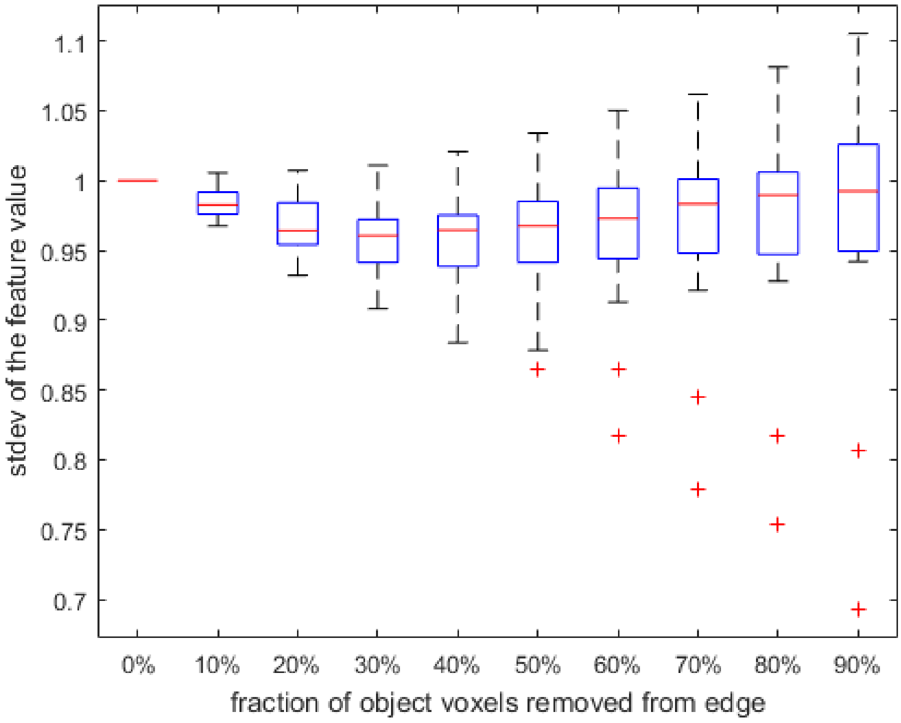

Figure S3 Relative scaling of AFS variance, as a function of the fraction of the edge voxels that are eroded prior to AFS estimation.

Supplementary Information S2: Pore space model

We model sub-resolution pores as idealized pulses of identical contrast H, cfr. Figure S4. The width W of these pulses, characteristic for the material's porosity, is our feature of interest.

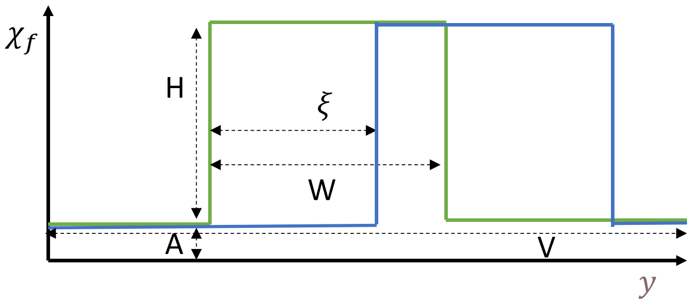

Figure S4 Idealized model for a single pore green in terms of the fine part of the materials' complex refractive index (green), as well as a shifted version (blue) by some correlation length ξ.

Evaluating equations (1) and (2) in terms of this model to obtain its DFEC reveals a piecewise linear function in terms of the correlation length ξ. For correlation lengths below the smallest pore size $\xi < W$, we find that the DFEC is proportional to:

$$DFEC \sim \frac{(V-W)A^2}{V} + \frac{W(A+H)^2}{V} - \frac{(V-W)A^2}{V} + \frac{\xi A^2}{V} - \frac{W(A+H)^2}{V} + \frac{\xi(A+H)^2}{V} - \frac{2\xi(A+H)A}{V},$$

which simplifies as:

$$DFEC \sim \xi\frac{H^2}{V}$$

On the other hand, for correlation lengths that exceed the largest pore size, i.e. $\xi \geq W$, we get:

$$DFEC \sim \frac{(V-W)A^2}{V} + \frac{W(A+H)^2}{V} - \frac{(V-2W)A^2}{V} - \frac{2WA(A+H)}{V}$$

Which simplifies to:

$$DFEC \sim \frac{WH^2}{V}$$

In other words, the $DFEC$ values form, by approximation, a piecewise linear function of ξ that starts with a sloped segment and transitions into a constant segment, with the segment transition located around $\xi = W$.

A real-world sample consists of a variety of pores sizes with non-spherical shapes, which result in the autocorrelation function being a weighted average of apparent cross-sectional pore sizes. If the correlation length ξ is below the smallest pore size W_{min}, each contribution to the $DFEC$ will be in the sloped regime of the function, and thus the total $DFEC$ slope will be largest in magnitude. With increasing correlation length values ξ between W_{min} and the largest pore size W_{max} the slope would decrease until reaching 0 for $\xi = W_{max}$. Due to the quantized nature of such measurements in finite-sized voxels, the measured slope would therefore characterize a weighted average of typically encountered pore sizes and therefore be a characteristic for the pore size distribution. Furthermore, the slope may be robustly extracted from many sample points, i.e. dark field acquisitions at different correlation lengths ξ. This consideration is the motivation for extracting the slope as the characteristic feature.
